# Insights into chromosomal evolution and sex determination of *Pseudobagrus ussuriensis* (Bagridae, Siluriformes) based on a chromosome-level genome

**DOI:** 10.1093/dnares/dsac028

**Published:** 2022-07-21

**Authors:** Chuankun Zhu, Haiyang Liu, Zhengjun Pan, Lei Cheng, Yanhong Sun, Hui Wang, Guoliang Chang, Nan Wu, Huaiyu Ding, Haitao Zhao, Lei Zhang, Xiangsheng Yu

**Affiliations:** Jiangsu Key Laboratory for Eco-Agriculture Biotechnology Around Hongze Lake, Jiangsu Collaborative Innovation Center of Regional Modern Agriculture & Environmental Protection, Jiangsu Engineering Laboratory for Breeding of Special Aquatic Organisms, Huaiyin Normal University, Huai’an 223300, China; Key Laboratory of Tropical and Subtropical Fishery Resources Application and Cultivation, Ministry of Agriculture and Rural Affairs, Pearl River Fisheries Research Institute, Chinese Academy of Fishery Sciences, Guangzhou 510380, China; Jiangsu Key Laboratory for Eco-Agriculture Biotechnology Around Hongze Lake, Jiangsu Collaborative Innovation Center of Regional Modern Agriculture & Environmental Protection, Jiangsu Engineering Laboratory for Breeding of Special Aquatic Organisms, Huaiyin Normal University, Huai’an 223300, China; Key Laboratory of Freshwater Aquatic Biotechnology and Breeding, Ministry of Agriculture and Rural Affairs, Heilongjiang Fisheries Research Institute, Chinese Academy of Fishery Sciences, Harbin 150070, China; Wuhan Aquaculture Science Research Institute, Wuhan 430207, China; Jiangsu Key Laboratory for Eco-Agriculture Biotechnology Around Hongze Lake, Jiangsu Collaborative Innovation Center of Regional Modern Agriculture & Environmental Protection, Jiangsu Engineering Laboratory for Breeding of Special Aquatic Organisms, Huaiyin Normal University, Huai’an 223300, China; Jiangsu Key Laboratory for Eco-Agriculture Biotechnology Around Hongze Lake, Jiangsu Collaborative Innovation Center of Regional Modern Agriculture & Environmental Protection, Jiangsu Engineering Laboratory for Breeding of Special Aquatic Organisms, Huaiyin Normal University, Huai’an 223300, China; Jiangsu Key Laboratory for Eco-Agriculture Biotechnology Around Hongze Lake, Jiangsu Collaborative Innovation Center of Regional Modern Agriculture & Environmental Protection, Jiangsu Engineering Laboratory for Breeding of Special Aquatic Organisms, Huaiyin Normal University, Huai’an 223300, China; Jiangsu Key Laboratory for Eco-Agriculture Biotechnology Around Hongze Lake, Jiangsu Collaborative Innovation Center of Regional Modern Agriculture & Environmental Protection, Jiangsu Engineering Laboratory for Breeding of Special Aquatic Organisms, Huaiyin Normal University, Huai’an 223300, China; Jiangsu Key Laboratory for Eco-Agriculture Biotechnology Around Hongze Lake, Jiangsu Collaborative Innovation Center of Regional Modern Agriculture & Environmental Protection, Jiangsu Engineering Laboratory for Breeding of Special Aquatic Organisms, Huaiyin Normal University, Huai’an 223300, China; Key Laboratory of Fishery Sustainable Development and Water Environment Protection of Huai’an City, Huai’an Sub Center of the Institute of Hydrobiology, Chinese Academy of Sciences, Huai’an 223002, China; Huai’an Fisheries Technical Guidance Station, Huai’an 223001, China

**Keywords:** *Pseudobagrus ussuriensis*, whole-genome sequencing, chromosome evolution, sex-determining region, sex chromosome

## Abstract

*Pseudobagrus ussuriensis* is an aquaculture catfish with significant sexual dimorphism. In this study, a chromosome-level genome with a size of 741.97 Mb was assembled for female *P. ussuriensis*. A total of 26 chromosome-level contigs covering 97.34% of the whole-genome assembly were obtained with an N50 of 28.53 Mb and an L50 of 11. A total of 24,075 protein-coding genes were identified, with 91.54% (22,039) genes being functionally annotated. Based on the genome assembly, four chromosome evolution clusters of catfishes were identified and the formation process of *P. ussuriensis* chromosomes was predicted. A total of 55 sex-related quantitative trait loci (QTLs) with a phenotypic variance explained value of 100% were located on chromosome 8 (chr08). The QTLs and other previously identified sex-specific markers were located in a sex-determining region of 16.83 Mb (from 6.90 to 23.73 Mb) on chr08, which was predicted as the X chromosome. The sex-determining region comprised 554 genes, with 135 of which being differently expressed between males and females/pseudofemales, and 16 candidate sex-determining genes were screened out. The results of this study provided a useful chromosome-level genome for genetic, genomic and evolutionary studies of *P. ussuriensis*, and also be useful for further studies on sex-determination mechanism analysis and sex-control breeding of this fish.

## 1. Introduction

Siluriformes contains more than 3,000 known living species,^1^ which are commonly called catfishes. Karyotypic studies revealed that chromosome numbers of catfishes ranged from 2n = 24 in *Liobagrus marginatus* to 2n = 100 in *Clarias batrachus*, with 2n = 52 being the dominant type.^2^ In some cases, chromosome numbers even differ among different populations of the same species.^3^^,^^4^ As chromosome numbers showed continuous changes in the interval from 2n = 48 to 2n = 60, and Robertsonian translocation, chromosomal fission and fusion, and even the fourth round whole genome duplication were believed to occur during genome evolution of Siluriformes species,^5^^,^^6^ catfishes were considered as potential models for chromosomal and genomic evolutionary studies of teleost.^2^ Additionally, visible sex chromosomes were identified in a few species of the Clariidae and Amblycipitidae families,^2^ but not in most of other catfish families.

In bony fishes, the sex determination mechanisms are diverse, with transitions between different genetic and environmental determination systems, and often differ among close-related species and even different populations of the same fish species.^7^^,^^8^ Scholars have strong and persistent interests in studying the sex determination mechanisms and sex chromosomes of fishes, which can provide significant information for breeding through sex control and revealing the molecular and evolutionary mechanisms of vertebrate sex chromosomes.^9^^,^^10^ Previous karyotypic studies indicate that most fishes have morphologically undifferentiated homomorphic sex chromosomes, and about only 10% species contain heteromorphic sex chromosomes.^11^^,^^12^ The male heterogametic XX/XY and female heterogametic ZW/ZZ sex determination systems are the two most common categories in teleosts, although there are other systems, including XX/XO, ZO/ZZ and X1X1X2X2/X1X2Y.^12^ Compared with birds and mammals, sex chromosomes in fishes studied so far are believed to be much younger, which make them potential models to analyze the evolution and differentiation of sex chromosomes at the molecular level.^13^^,^^14^

For fishes without visible sex chromosomes, sex determination systems can be deduced using sex-specific markers.^15–17^ In recent years, different types of sex-specific markers including sequence characterized amplified regions (SCARs),^18^^,^^19^ single sequence repeats (SSRs),^20^^,^^21^ single nucleotide polymorphism (SNPs),^22^^,^^23^ restriction site-associated DNA (RAD)/2b-RAD tags,^17^^,^^24^ and quantitative trait loci (QTLs)^25^^,^^26^ have been identified in aquatic animals. Through mapping these sex-specific markers to genetic linkage maps, physical maps and assembled genomes, sex-determining regions have been identified in many fish species.^23^^,^^27^^,^^28^

Chromosome-level genomes are essential for better localizations of sex determination regions and identifications of potential sex-determining genes. With the development of sequencing technologies, the long-read third-generation sequencing technologies, including the Oxford Nanopore and the PacBio methods, are widely used and genome assemblies are continuously reported in aquatic animals in recent years. Through combining the third-generation sequencing method (Oxford Nanopore or PacBio) and the high-through chromosome conformation capture (Hi-C) technology, genomes can be assembled into the chromosome level, which have been successfully used in many fish species.^29–31^ Chromosome-level genomes have also been reported in catfishes, such as channel catfish (*Ictalurus punctatus*),^32^ yellow catfish (*Pelteobagrus fulvidraco*),^33^ giant devil catfish (*Bagarius yarrelli*),^34^ Southern catfish (*Silurus meridionalis*),^35^ Chinese longsnout catfish (*Leiocassis longirostris*),^36^ striped catfish (*Pangasianodon hypophthalmus*),^37^ and redtail catfish (*Hemibagrus wyckioides*).^38^ These chromosome-level assemblies have been widely used as reliable reference genomes for studies of many fields, including ecological adaptation,^35^^,^^39^^,^^40^ genome evolution,^41–43^ gene evolution,^44^^,^^45^ immunology,^46–48^ and identification of target-trait-related genes,^49^^,^^50^ in aquatic animals. Moreover, chromosome-level genomes have also been used for studies on sex determination mechanisms, including identification of sex determination regions and genes,^51–53^ sex reversal,^54^^,^^55^ and sex chromosome evolutions.^10^^,^^14^^,^^56^

The Ussuri catfish *Pseudobagrus ussuriensis* belongs to the Bagridae family of Siluriformes. This catfish naturally distributes from the northern Heilongjiang River basin to the southern Pearl River basin of China.^57^ In recent decades, due to overfishing, habitat destruction and water pollution, natural resources of *P. ussuriensis* have sharply declined, making it a rare species in its natural distribution areas.^58^ In order to protect and sustainably use natural resources, artificial reproduction, culturing and breeding have been performed in this fish, and it is now cultured in more than one third provinces of China. Additionally, the sexual dimorphism in *P. ussuriensis* is extremely obvious,^19^ with males growing approximately three times faster and larger than females, according to our measurement data of 2-year-old individuals. Given the growth advantages of males, all-male breeding through artificial sex control is a promising approach to increase the aquaculture yields of *P. ussuriensis*.

Previous studies of *P. ussuriensis* mainly focused on artificial reproduction and culture,^59^^,^^60^ interspecific hybridization breeding,^61^^,^^62^ and nutrition.^63^ Although studies are increasing in recent years, available genetic and genomic information of this fish is still limited, and studies on its sex-determination mechanism are seldom reported. According to our previous studies, the sex determination system of *P. ussuriensis* was preliminarily deduced as the XX/XY type based on two validated male-specific markers,^19^^,^^21^ and more male-specific tags were also identified.^64^ Nevertheless, our understandings on the sex determination mechanism of *P. ussuriensis* are still at the preliminary level. In the present study, a chromosome-level genome of *P. ussuriensis* was intended to be assembled using a combined method of Oxford Nanopore, Illumina, and Hi-C technologies, and the chromosomal evolution of *P. ussuriensis* was planned to analyze based on the genome assembly. The sex determination region was intended to be identified through mapping newly detected sex-related QTLs and previously identified sex-specific markers to the assembled genome. The results obtained in this study will be useful for genetic, genomic, and evolutionary studies, as well as sex-determination mechanism analysis and sex-control breeding of *P. ussuriensis*.

## 2. Materials and methods

### 2.1. Sample preparation

All applicable international, national, and/or institutional guidelines for the care and use of animals were strictly followed. All animal sample collection protocols complied with the current laws of China. Fish used in this study were provided by Huai’an Fisheries Technical Guidance Station and were reared in tanks of the Jiangsu Engineering Laboratory for Breeding of Special Aquatic Organisms, Huai’an, China. An adult female, which was identified through gametes, was randomly selected in May 2020 for genome sequencing. The muscle tissue of this fish was collected and stored in liquid nitrogen for following experiments. Total genomic DNA was extracted using a QIAGEN^®^ Genomic DNA extraction kit (Qiagen, Germany), and the DNA quality was measured using a NanoDrop™ One UV-Vis spectrophotometer (Thermo, USA), and was quantified using a Qubit^®^ 3.0 fluorometer (Invitrogen, USA).

### 2.2. Library construction and sequencing

The nanopore library was constructed using an Oxford Nanopore Ligation Sequencing kit SQKLSK109 (Oxford Nanopore Technologies, UK) following the manufacturer’s instructions. Briefly, size-selection (10–50 kb) was initially performed using ∼10 μg of gDNA by a BluePippin pulsed-field gel apparatus (Sage Science, USA). After damage and end repairs, adenines were added at the ends of DNA segments and then adapters were ligated to finish the construction of the nanopore library. After testing using a Qubit^®^ 3.0 fluorometer (Invitrogen), the library was sequenced on R9.4 Flowcell using a PromethION sequencer (Oxford Nanopore Technologies, UK) at NextOmics Bioscience Co., Ltd (Wuhan, China). Then the ONT software Albacore v1.2.665 was used for base calling on fast5 files, and reads passed the filtration were considered as high-quality data, which were used for further analyses.

The Illumina library, which was used for polishing of the assembled nanopore genome, was constructed using a Truseq Nano DNA HT Sample Preparation Kit (Illumina, USA) according to the manufacturer’s recommendations. In brief, the genomic DNA was fragmented by sonication into short segments with a size of 350 bp. Then DNA segments were end polished, A-tailed, ligated with Illumina sequencing adapters and amplified through PCR. After purification (using the AMPure XP system) of PCR amplicons, the library was analyzed for size distribution using an Agilent2100 Bioanalyzer and quantified through real-time PCR. Finally, the library was sequenced by an Illumina NovaSeq platform at NextOmics Bioscience Co., Ltd (Wuhan) though 150 bp paired-end sequencing.

The Hi-C library was prepared according to a previously reported procedure^66^ with slight modifications. Firstly, ∼100 mg of muscle sample was shattered and treated with nuclei isolation buffer supplemented with 2% formaldehyde to induce crosslinking. Secondly, 2.5 M glycine was added to stop the crosslinking reaction. After purification, the nuclei were digested using 100 units of *DpnII* restriction enzyme and marked with biotin-14-dCTP, and then ligated with T4 DNA Ligase. The ligated DNA was sheared into fragments with a size range of 300–600 bp, and then these fragments were blunt-end repaired and A-tailed, followed by purification through biotin–streptavidin-mediated pull down. Finally, after quantification, the Hi-C library was sequenced using an Illumina Hiseq X Ten platform (Illumina) at NextOmics Bioscience Co., Ltd (Wuhan) though 150 bp paired-end sequencing.

A transcriptome library used for gene annotation of the *P. ussuriensis* genome was constructed using 11 tissues, including liver, spleen, heart, kidney, gills, brain, pituitary, muscle, skin, gonads, and intestine, from the fish used for genome sequencing. Total RNA of each tissue was extracted using TRIzol Reagent Kit (Invitrogen), according to the manufacturer’s protocol. Then 100 ng RNA from each tissue was pooled together for the cDNA library construction. The NEBNext^®^ Ultra™ RNA Library Prep Kit for Illumina^®^ (NEB, USA) was used to construct the library following the manufacturer’s protocol. Finally, the 150 bp paired-end sequencing was performed for the library through a high-throughput Illumina HiSeq 4000 platform at Novogene Co., Ltd., Beijing, China, following the manufacturer’s instructions (Illumina).

### 2.3. Genome assembly

Before assembly, the filtered Oxford Nanopore sequencing data were corrected by Nextdenovo (https://github.com/Nextomics/NextDenovo, 10 May 2021, date last accessed), using the following parameters: read_cuoff = 1 k, seed_cutoff = 10 k, blocksize = 2 g. Then, the WTDBG v1.2.8 (WTDBG, RRID: SCR_017225) software was used for genome assembly (parameters settings: wtdbg-1.2.8 -k 0 -p 17 -S 2; kbm-1.2.8 -k 0 -p 15 -S 2 -O 0; wtdbg-cns -k 13 -c 3). The assembled contigs were polished through three rounds of consensus correction by BWA v0.7.1267 (BWA, RRID: SCR 010910) and Pilon^68^ (Pilon, RRID: SCR 014731) using Illumina reads.

Raw Hi-C data were trimmed using HiC-Pro (v2.8.0) according to the reported method.^69^ Then Bowtie2 (v2.3.2)^70^ was used to map the clean paired-end reads to the draft assembled contigs. Both reads of the paired-end data were mapped independently, and only read pairs with both ends being uniquely aligned to the assembled contigs were used for further analysis. The valid Hi-C reads were used to assemble the chromosomal-level genome using LACHESIS^69^ (parameters: CLUSTER MIN RE SITES = 100, CLUSTER MAX LINK DENSITY = 2.5, LUSTER NONINFORMATIVE RATIO = 1.4, ORDER MIN N RES IN TRUNK = 60, and ORDER MIN N RES IN SHREDS = 60) through clustering, ordering and orienting of contigs. Finally, a genome-wide Hi-C heatmap was constructed through ggplot2 in the R package to evaluate the quality of the assembled chromosomal-level genome, and genome characters were plotted using Circos v0.69-6.71

### 2.4. Annotations of repeat sequences and non-coding RNAs

Tandem repeat elements were searched using tandem repeats finder v4.07b^72^ with default parameters throughout the genome, and SSRs were identified using the software GMATA v2.2.73 All tandem repeats were softmasked before the identification of transposable elements (TE), which were performed through combining the *de novo* and homology-based methods. In brief, a *de novo* repeat library was initially constructed using MITE-hunter^74^ and RepeatModeler v1.0.1175 with default parameter settings. Then the library was aligned against the TEclass Repbase^76^ to classify TEs into different repeat families. Finally, the software RepeatMasker v1.331^75^ was used to search both known and novel TEs by mapping sequences against the *de novo* repeat library and Repbase TE library, and overlapped TEs belonging to the same repeat class were combined.

Two strategies, which were searching against database and prediction with model, were used to identify non-coding RNAs (ncRNAs) in the assembled genome. Transfer RNAs (tRNAs) were predicted using the software tRNAscan-SE v2.077 with eukaryote parameters. The Rfam database^78^ was applied to detect MicroRNAs (miRNAs), ribosomal RNAs (rRNAs), small nuclear RNAs (snRNAs), and small nucleolar RNAs (snoRNAs) using cmscan of the software Infernal v1.1.2.79 Additionally, the software RNAmmer v1.280 was used to predict rRNAs and their subunits.

### 2.5. Gene predictions and functional annotations

All repeat sequences of the assembled genome were masked before gene prediction. The homology search, reference guided transcriptome assembly and *de novo* prediction approaches were independently used for gene predictions. For the homolog prediction method, protein sequences of five fishes, including yellow catfish (*P. fulvidraco*) (http://gigadb.org/dataset/100506, 15 June 2021, date last accessed), channel catfish (*I. punctatus*) (https://www.ncbi.nlm.nih.gov/genome/198, 15 June 2021, date last accessed), grass carp (*Ctenopharyngodon idellus*) (http://www.ncgr.ac.cn/grasscarp/, 15 June 2021, date last accessed), zebrafish (*Danio rerio*) (https://www.ncbi.nlm.nih.gov/genome/?term=zebrafish, 15 June 2021, date last accessed), and medaka (*Oryzias latipes*) (https://www.ncbi.nlm.nih.gov/genome/?term=medaka, 15 June 2021, date last accessed), were downloaded from public databases. Then these sequences were aligned to the genome assembly of *P. ussuriensis* using GeMoMa v1.6.181 to detect homologous peptides. For the RNA-seq-based prediction approach, raw reads were filtered using fastp v0.12.682 and clean reads were aligned to the genome assembly using STAR v2.7.3a.^83^ The transcripts were then assembled using Stringtie v1.3.4d^84^ and open reading frames were predicted using PASA v2.3.3.85 Using predicted genes of the RNA-seq-based method as the training set, Augustus v3.3.186 was applied for *de novo* gene prediction with default parameters. Finally, an integrated gene set was produced by EVidenceModeler v1.1.1,85 and genes being homologous to transposons were filtered using TransposonPSI v08222010 (http://transposonpsi. sourceforge.net/, 10 May 2021, date last accessed) and the miscoded genes were also removed. Furthermore, based on RNA-seq assemblies, untranslated regions and alternative splicing regions were determined using PASA. The accuracy of gene prediction and completeness of the genome assembly were assessed using Benchmarking Universal Single-Copy Orthologs v3.0.1 (BUSCO)^87^ by searching the predicted genes in the assembly for the conserved single-copy genes in Actinopterygii.

Five public databases, including NCBI non-redundant protein sequence database (NR), SwissProt, Clusters of orthologous groups for eukaryotic complete genomes (KOG), Kyoto Encyclopedia of Genes and Genomes (KEGG) and Gene Ontology (GO), were used for functional annotation of predicted protein-coding genes. Putative domains and GO terms of these genes were identified using InterProScan v5.32-71.0,88 while BLASTp v2.7.1 was used to compare the protein sequences of the *P. ussuriensis* genome against the remaining four public databases with an E value criterion of 1e^−5^. Finally, annotation results from the five databases were integrated to obtain the final gene set.

### 2.6. Phylogenetic analyses of the *P. ussuriensis* genome

Genome data of 10 teleosts, including *P. fulvidraco*, *I. punctatus*, *D. rerio*, *O. latipes*, *Astyanax mexicanus* (https://www.ncbi.nlm.nih.gov/genome/13073?genome_assembly_id=334665, 15 June 2021, date last accessed), *Takifugu rubripes* (https://www.ncbi.nlm.nih.gov/genome/63, 15 June 2021, date last accessed), *Oreochromis niloticus* (https://www.ncbi.nlm.nih.gov/genome/?term=Oreochromis+niloticus, 15 June 2021, date last accessed), *Monopterus albus* (https://www.ncbi.nlm.nih.gov/genome/24053?genome_assembly_id=302095, 15 June 2021, date last accessed), *Glyptosternum maculatum* (http://gigadb.org/dataset/100489, 15 June 2021, date last accessed) and *S. meridionalis* (https://www.ncbi.nlm.nih.gov/genome/9650, 15 June 2021, date last accessed), and an outgroup species *Latimeria chalumnae* (https://www.ncbi.nlm.nih.gov/genome/?term=coelacanth, 15 June 2021, date last accessed) were downloaded from open-source databases to identify gene families for the *P. ussuriensis* genome using OrthoMCL v2.0.9.89 Through the all-to-all BLASTP method with an E-value cutoff of 1e^−5^, protein-sequence similarities of the longest transcript were determined between *P. ussuriensis* and other species for all genes, which were then classified into three groups: orthologs, paralogs, and single copy orthologs.

The phylogenetic relationships between *P. ussuriensis* and another 11 fishes were analyzed using the single-copy orthologs shared by them. In brief, multiple alignments were performed through Mafft v7.31390 for each ortholog group, using their coding sequences. After elimination of poorly aligned sequences by Gblocks 0.91b,^91^ a phylogenetic tree was constructed using the GTRGAMMA model of RAxML v8.2.1092 with a bootstrap replicate of 1,000 times. Finally, the phylogenetic tree was visualized using MEGA v10.1.8.

Based on the phylogenetic tree, the MCMCTree software of the PAML v4.8 package^93^ was used to estimate divergent times between *P. ussuriensis* and the other 11 fish species. Four fossil calibration times, including divergence times between *D. rerio* and *I. punctatus* (126–179 Mya), *D. rerio* and *O. latipes* (205–255 Mya), *O. latipes* and *O. niloticus* (87–151 Mya), and *O. latipes* and *L. chalumnae* (425–446 Mya), were obtained from the TimeTree database (http://www.timet ree.org/, 15 June 2021, date last accessed) as the time control. Based on the divergent time tree, expansions and contractions of gene families were analyzed using the CAFE software.^94^

### 2.7. Comparative genomic analysis

To analyze chromosomal evolution of the *P. ussuriensis* (Bagridae) genome, syntenic comparisons were performed between *P. ussuriensis* and another four fishes of Siluriformes, including *P. fulvidraco* (Bagridae), *I. punctatus* (Ictaluridae), *S. meridionalis* (Siluridae), and *P. hypophthalmus* (Pangasiidae). Alignments of entire genomic DNA sequences between *P. ussuriensis* and other fishes were performed through Mummer v3.0,95 and syntenic relationships were plotted using the JCVI package.^96^

### 2.8. Identification of the sex-determination region

The correspondences between linkage groups (LGs) of the genetic linkage map^97^ and chromosomes (chrs) of the genome assembly were determined through mapping the SNP-containing 2b-RAD tags from the linkage map to the chromosomal-level genome using the Bowtie v2.4.0 software.^98^ The sex of individuals used for the genetic linkage map construction of *P. ussuriensis*^97^ were identified using the previously identified male-specific microsatellite marker *PuGT54*.^21^ Then sex-related QTLs were identified through MapQTL 6.099 using the previously reported method.^97^ Only the QTLs with phenotypic variance explained (PVE) values of 100% were used for further analyses.

The detected sex-related QTLs and sex-specific 2b-RAD tags identified in a previous study^64^ were mapped to the assembled *P. ussuriensis* genome using Bowtie v2.4.0. Moreover, male-specific markers identified in our previous studies^19^^,^^21^ were also mapped on the genome using Local BLASTn with an E-value cutoff of 1e^−10^. The chromosomal region containing more than 90% of the above segments was considered as the sex-determination region, and the chromosome containing the sex-determination region was predicted to be the sex chromosome of *P. ussuriensis*. Sequences of genes in the sex-determination region were compared with 15,495 differently expressed genes (DEGs) between gonads of males and females, as well as 14,022 DEGs between males and estradiol-treated males (pseudofemales), which were identified in a previous study.^100^ The genes corresponded to the DEGs that were shared between the two datasets were used for KOG, GO, and KEGG classifications to detect potential sex-determining genes.

## 3. Results

### 3.1. Genome sequencing and assembly

A total of 38.7 Gb Illumina clean data were obtained and used for estimations of the size and heterozygosity of the *P. ussuriensis* genome. The estimated genome size was 633.69 Mb, and the heterozygosity was 0.32%. Nanopore sequencing resulted in 75.0 Gb clean data, which were preliminarily assembled into 1,142 contigs. The assembled genome size was 741.89 Mb, with the N50 length of 5.61 Mb, the longest contig of 24.25 Mb, and the average contig length of 6.50 Mb (Table 1). The guanine–cytosine (GC) analysis resulted in an average GC content of 39.33% and a unimodal GC content distribution for the *P. ussuriensis* genome. The completeness of the genome assembly was evaluated to be 94.6% by BUSCO.

**Table 1.** Genome assembly statistics of *Pseudobagrus ussuriensis*

	Nanopore	Hi-C
Total contigs	1142	819
Total base (bp)	741,894,228	741,974,678
Total *N* (bp)	0	80,450
Average length (bp)	649,644	905,951
Longest contig (bp)	24,251,496	44,389,119
Shortest contig (bp)	1032	1000
L50	42	11
N50	5,612,645	28,525,251
L75	98	18
N75	1,908,622	22,812,222
L90	207	23
N90	482,052	18,557,309
GC (%)	39.33	39.33
Complete BUSCOs	4335 (94.6%)	3544 (97.4%)
Complete and single-copy BUSCOs	4163 (90.8%)	3484 (95.7%)
Complete and duplicated BUSCOs	172 (3.8%)	60 (1.6%)
Fragmented BUSCOs	83 (1.8%)	24 (0.7%)
Missing BUSCOs	166 (3.6%)	72 (2.0%)
Total BUSCO groups searched	4584	3640

**Figure 1.**
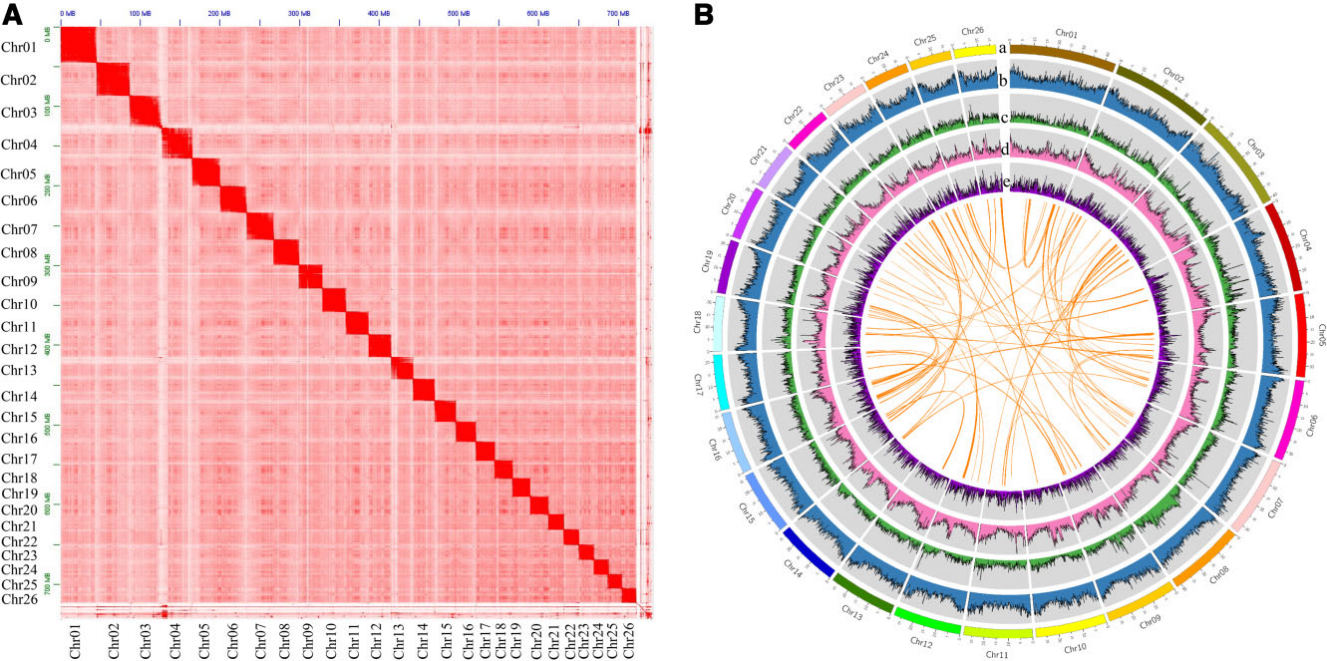
Genome assembly of *Pseudobagrus ussuriensis*. (A) The chromosomal-crosstalk heatmap based on Hi-C data. The blocks represent the contacts between one location and another. The color reflects the intensity of each contact, with deeper colors representing higher intensity. (B) Genome characteristics of *P. ussuriensis*, from outer to inner circles: a, the 26 chromosomes at the Mb scale; b–e, GC content, SNP density, repeat density and gene density across the genome, respectively, drawn in 1 Mb nonoverlapping windows.

The Hi-C sequencing resulted in 78.60 Gb clean data, containing 524,470,464 clean paired-end reads, among which, 164.62 million unique mapped reads and 118.88 million valid reads (45.33% of clean reads and 72.21% of unique mapped reads) were obtained. Using the Hi-C data, 819 contigs with a size of 741.97 Mb were assembled, and the N50 and average contig lengths were 28.53 and 9.06 Mb (Table 1), respectively. The genome assembly was anchored and orientated into 26 chromosomes with a size range of 17.45–44.39 Mb and a total size of 722.22 Mb, which covered 97.34% of the whole assembled genome (Table 2). The GC contain of the Hi-C genome assembly was 39.33% (Table 1), which was identical with the nanopore assembly. The result of BUSCO estimation showed that the completeness of the Hi-C genome assembly was 97.4% (Table 1). The chromosomal-crosstalk heatmap indicated the complete and robust assembly of the *P. ussuriensis* genome (Fig. 1A), and characteristics of this genome assembly were also analyzed (Fig. 1B).

**Table 2.** Correspondence between the physical genome and the genetic linkage map of *Pseudobagrus ussuriensis*

Chromosome	Linkage group	Physical length (Mb)	Genetic size (cM)	No. of genes	Gene density (genes/Mb)
chr01	LG4	44.39	100.28	1555	35.03
chr02	LG3	42.31	121.38	1376	32.52
chr03	LG2	40.92	134.97	1231	30.08
chr04	LG5	37.81	103.10	1210	32.00
chr05	LG8	34.58	85.47	1074	31.06
chr06	LG7	33.77	103.94	1058	31.33
chr07	LG9	33.32	102.64	1090	32.71
chr08	LG1	32.06	108.52	912	28.45
chr09	LG6	29.58	116.98	967	32.69
chr10	LG13	29.44	91.20	751	25.51
chr11	LG12	28.53	151.36	978	34.28
chr12	LG20	28.21	106.71	965	34.21
chr13	LG11	27.56	110.15	648	23.51
chr14	LG23	26.75	109.60	860	32.15
chr15	LG10	26.48	87.00	868	32.78
chr16	LG15	25.77	84.26	805	31.24
chr17	LG14	22.93	118.50	879	38.33
chr18	LG16	22.81	98.47	822	36.04
chr19	LG21	22.37	90.78	848	37.91
chr20	LG22	22.30	92.32	881	39.51
chr21	LG19	19.40	79.15	649	33.45
chr22	LG25	19.03	105.93	681	35.79
chr23	LG18	18.56	93.55	559	30.12
chr24	LG24	18.43	96.61	684	37.11
chr25	LG17	17.46	82.53	633	36.25
chr26	LG26	17.45	81.31	680	38.97
Total		722.22	2656.72	23664	32.77
Coverage rate (%)		97.34	98.93		

### 3.2. Genome annotation

A total of 2,477,914 repeat sequences were identified with a size of 296,239,694 bp, which accounted for 39.93% of the *P. ussuriensis* genome, and TEs were the most abundant class with a percentage of 32.99% (Supplementary Table S1). Identification of ncRNAs resulted in 14,393 sequences, including 10,755 tRNAs, 337 miRNAs, 312 rRNAs, and 216 snRNAs (Supplementary Table S2).

Among the three gene-prediction methods, the homolog method resulted in 34,685 genes (Table 3), the RNA-seq method identified 18,081 genes, and the *de novo* method predicted 26,128 genes (Table 3). Through integration of the three gene sets, a total of 24,075 genes with an average length of 14,909 bp and an average CDS length of 1,657 bp (Table 3) were finally predicted for the *P. ussuriensis* genome. There were ∼10 exons for these genes on average, and the average length of exons was 164 bp with the average intron length of 1,451 bp (Table 3). The length distribution trends of gene, CDS, exon and intron, as well as number distributions of exon and intron were shown in Supplementary Fig. S1. Chromosome 1 (chr1) contained the most genes (1,555), while chr25 had the fewest (633), and the gene density was the highest in chr20 (39.51 genes/Mb) and lowest in chr13 (23.51 genes/Mb, Table 2). The 26 chromosomes comprised 23,664 (98.29%) of the 24,075 genes (Table 2), with the other 411 genes being located on unanchored contigs. According to the BUSCO assessments, 94.51% complete BUSCO genes were successfully predicted, which comprised 92.66% of complete single-copy BUSCOs (S) and 1.84% of complete and duplicated BUSCOs (D) from the 3,640 assessed genes (Supplementary Table S3). The results of BUSCO estimations indicated that the assembly and annotation of the *P. ussuriensis* genome were high in integrity and quality. Additionally, through annotations in five public databases, 22,039 (91.54%) of the predicted genes were annotated in at least one database (Supplementary Table S4). The assembly and annotation data of *P. ussuriensis* were compared with those of some reported catfishes (Table 4), including *P. fulvidraco*,^33^*L. longirostris*,^36^*H. wyckioides*,^38^*G. maculatum*,^101^*B. yarrelli*,^34^*S. meridionalis*,^35^*Silurus asotus*,^102^*Silurus glanis*,^103^*Clarias batrachus*,^104^*I. punctatus*,^105^ and *P. hypophthalmus*.^37^

**Table 3.** Gene prediction of the *Pseudobagrus ussuriensis* genome

		No. of genes	Average gene length (bp)	Average CDS length (bp)	Average No. of exons per gene	Average exon length (bp)	Average intron length (bp)
RNA-seq	NGS	18,081	21,022.24	3,781.77	11.09	340.99	1,708.55
Predicted	AUGUSTUS	26,128	13,012.66	1,570.09	9.73	161.38	1,310.86
	PASA	16,838	17,872.69	3,804.68	11.15	341.2	1,385.87
Homology	*Pelteobagrus fulvidraco*	57,395	21,779.86	1,264.01	6.72	188.2	3,589.04
	*Ictalurus punctatus*	46,565	28,501.24	1,620.15	9.18	176.58	3,288.14
	*Danio rerio*	136,807	18,850.56	1,591.23	6.79	234.31	2,980.32
	*Oryzias latipes*	53,967	44,948.92	1,683.61	9.49	177.4	5,095.86
	*Ctenopharyngodon idellus*	75,906	10,971.60	1,106.53	3.83	289.03	3,487.81
Total	Evidence modeler	24,075	14,909.21	1,656.70	10.13	163.5	1,451.06

**Table 4.** Comparisons between the *Pseudobagrus ussuriensis* genome and assembled genomes of other Siluriformes species

Species	Family	Assembly size (Mb)	Number of chrs	Contig N50 (Mb)	Genome coverage (%)	GC (%)	Identified genes	Annotated genes	Repetitive elements (%)	Sources
*Pseudobagrus ussuriensis*	Bagridae	741.97	26	28.53	97.34	39.33	24,075	22,039	39.93	This study
*Pelteobagrus fulvidraco*	Bagridae	732.80	26	25.80	94.20	39.60	24,552	24,552	38.47	Gong *et al*.^33^
*Leiocassis longirostris*	Bagridae	703.19	26	28.03	97.44	39.67	23,708	23,170	33.99	He *et al*.^36^
*Hemibagrus wyckioides*	Bagridae	789.79	29	22.08	97.70	–	22,794	21,142	40.12	Shao *et al*.^38^
*Glyptosternon maculatum*	Sisoridae	704.80	24	27.80	91.70	39.60	22,066	20,234	32.76	Xiao *et al*.^101^
*Bagarius yarrelli*	Sisoridae	571.00	–	3.10	95.30	–	19,027	17,740	35.26	Jiang *et al*.^34^
*Silurus meridionalis*	Siluridae	738.90	29	28.04	99.60	39.00	22,965	22,519	40.12	Zheng *et al*.^35^
*Silurus asotus*	Siluridae	744.12	29	28.82	94.00	–	22,894	–	29.80	Chen *et al*.^102^
*Silurus glanis*	Siluridae	793.40	–	3.20	84.20	39.20	21,316	20,532	39.50	Ozerov *et al*.^103^
*Clarias batrachus*	Clariidae	821.00	–	0.36	95.20	39.20	22,914	22,587	30.30	Li *et al*.^104^
*Ictalurus punctatus*	Ictaluridae	845.00	29	7.73	96.80	39.69	21,556	20,370	32.56	Chen *et al*.^105^
*Pangasianodon hypophthalmus*	Pangasiidae	731.70	30	29.50	98.46	38.89	18,895	18,604	36.90	Gao *et al*.^37^

### 3.3. Genomic evolution analysis

**Figure 2.**
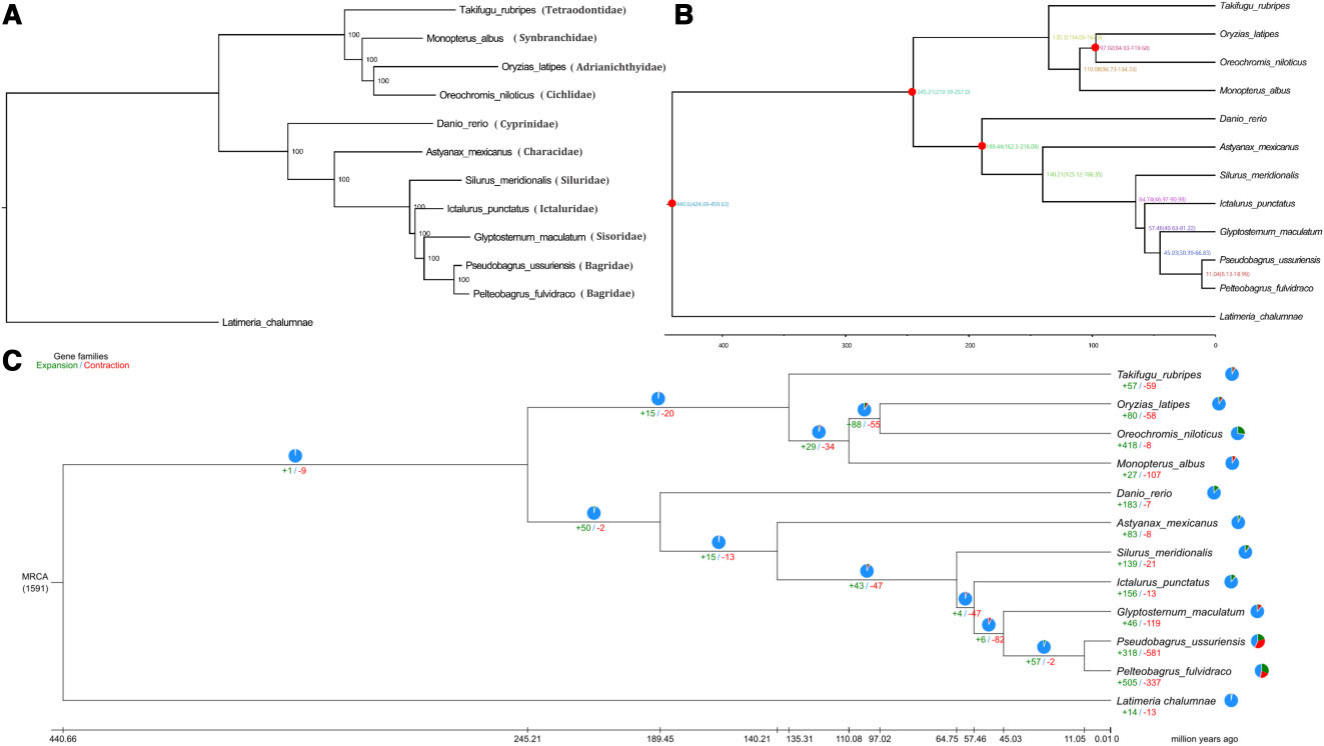
Phylogenomic trees between *Pseudobagrus ussuriensis* and other fishes. (A) Phylogenetic tree between *P. ussuriensis* and another 11 fishes. (B) Divergence time tree between *P. ussuriensis* and another 11 fish species. Numbers on the branches represent estimated diverge times in millions of years ago, and nodes marked with red circles indicate the calibration time from fossil. (C) Expansion and contraction of gene families in *P. ussuriensis*. MRCA stands for the most recent common ancestor; pie charts and numbers represent the proportion and numbers of gene families with expansion marked with green and contraction marked with red, respectively.

Results of gene family clustering with other teleost showed that 21,150 (87.85%) genes, including 2,698 single copy orthologs and 556 unique genes, in the *P. ussuriensis* genome could be assigned into 15,636 gene families with 152 of which being unique in *P. ussuriensis* (Supplementary Table S5). Using the 2,698 single copy orthologs, a phylogenetic tree was constructed between *P. ussuriensis* and another 11 fish species. The phylogenetic tree showed that *P. ussuriensis* firstly clustered with *P. fulvidraco* (Bagridae), and then successively clustered with *G. maculatum* (Sisoridae), *I. punctatus* (Ictaluridae), and *S. meridionalis* (Siluridae) forming the Siluriformes clade (Fig. 2A). Then the Siluriformes clade formed the Ostariophysan fish clade with *A. mexicanus* (Characiformes) and *D. rerio* (Cypriniformes) (Fig. 2A). Based on fossil calibration times of known fishes, ancestors of *P. ussuriensis* and *P. fulvidraco* have separated in ∼11.04 Mya (Fig. 2B). The results of gene family expansions and contractions showed that 318 gene families experienced expansions and 581 underwent contractions (Fig. 2C).

**Figure 3.**
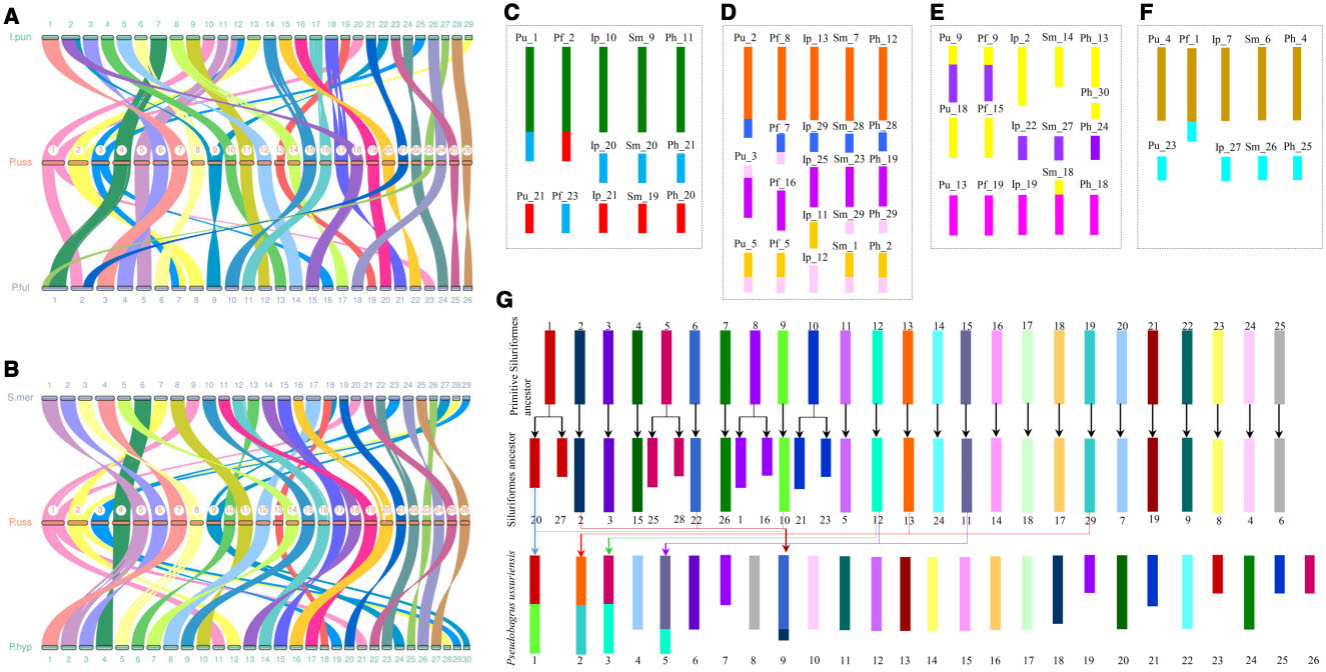
Chromosomal syntenic relationships and estimated chromosome evolution processes between *Pseudobagrus ussuriensis* and another four catfishes. (A) The syntenic relationships among chromosomes of *P. ussuriensis* (P. uss), *Pelteobagrus fulvidraco* (P. ful), and *Ictalurus punctatus* (I. pun). (B) The syntenic relationships among chromosomes of *P. ussuriensis* (P. uss), *Silurus meridionalis* (S. mer), and *Pangasianodon hypophthalmus* (P. hyp). (C)–(F) Estimated catfish chromosome evolution clusters 1–4, Pu *P. ussuriensis*, Pf *P. fulvidraco*, Ip *I. punctatus*, Sm *S. meridionalis*, and Ph *P. hypophthalmus*. (G) The estimated evolution process of 26 chromosomes in *Pseudobagrus ussuriensis*.

Syntenic relationships of the *P. ussuriensis* chromosomes with four catfish species were analyzed through genomic comparisons. The results between genomes of *P. ussuriensis* and *P. fulvidraco* showed that 20 chrs of *P. ussuriensis* (Pu_chr) exhibited a one-to-one relationship with chrs of *P. fulvidraco* (Pf_chr), while the remaining six chrs involved three one-to-two relationships (Pu_chr1 to Pf_chr2 and Pf_chr23, Pu_chr2 to Pf_chr7 and Pf_chr8, and Pu_chr3 to Pf_chr7 and Pf_chr16) and two two-to-one relationships (Pu_chr4 and Pu_chr23 to Pf_chr1, and Pu_chr1 and Pu_chr21 to Pf_chr2) (Fig. 3A). Comparisons between Pu_chrs and chrs of *I. punctatus* (Ip_chr) resulted in 20 one-to-one relationships, five one-to-two relationships (Pu_chr1 to Ip_chr10 and Ip_chr20, Pu_chr2 to Ip_chr13 and Ip_chr29, Pu_chr3 to Ip_chr12 and Ip_chr25, Pu_chr5 to Ip_chr11 and Ip_chr12, and Pu_chr9 to Ip_chr2 and Ip_chr22), and one two-to-one relationship (Pu_chr9 and Pu_chr18 to Ip_chr2) (Fig. 3A). Syntenic relationships between Pu_chrs and chrs of *S. meridionalis* (Sm_chr) included 21 one-to-one, four one-to-two (Pu_chr1 to Sm_chr9 and Sm_chr20, Pu_chr2 to Sm_chr7 and Sm_chr28, Pu_chr3 to Sm_chr23 and Sm_chr29, and Pu_chr9 to Sm_chr18 and Sm_chr27) and one two-to-one (Pu_chr9 and Pu_chr13 to Sm_chr18) relationships (Fig. 3B). The comparison results between Pu_chrs and chrs of *P. hypophthalmus* (Ph_chr) showed 22 one-to-one relationships and four one-to-two relationships (Pu_chr1 to Ph_chr11 and Ph_chr21, Pu_chr2 to Ph_chr12 and Ph_chr28, Pu_chr3 to Ph_chr19 and Ph_chr29, and Pu_chr9 to Ph_chr24 and Ph_chr30) (Fig. 3B).

Through the chromosomal syntenic relationships between *P. ussuriensis* and another four catfish species, four evolutionary chromosome clusters (Fig. 3C–F) of the five catfishes were identified, which caused different chromosome numbers of the five fishes. The first cluster involved two chromosomes from *P. ussuriensis* (Pu_chr1 and Pu_chr21) and *P. fulvidraco* (Pf_chr2 and Pf_chr23), and three chromosomes from *I. punctatus* (Ip_chr10, Ip_chr20 and Ip_chr21), *S. meridionalis* (Sm_chr9, Sm_chr19 and Sm_chr20), and *P. hypophthalmus* (Ph_chr11, Ph_chr20 and Ph_chr21) (Fig. 3C). The second cluster was the most complex one which contained three chromosomes of *P. ussuriensis* (Pu_chr2, Pu_chr3 and Pu_chr5), four of *P. fulvidraco* (Pf_chr5, Pf_chr7, Pf_chr8, and Pf_chr16) and five of *I. punctatus* (Ip_chr11, Ip_chr12, Ip_chr13, Ip_chr25 and Ip_chr29), *S. meridionalis* (Sm_chr1, Sm_chr7, Sm_chr23, Sm_chr28 and Sm_chr29) and *P. hypophthalmus* (Ph_chr2, Ph_chr12, Ph_chr19, Ph_chr28 and Ph_chr29) (Fig. 3D). The third cluster included three chromosomes of *P. ussuriensis* (Pu_chr9, Pu_chr13 and Pu_chr18), *P. fulvidraco* (Pf_chr9, Pf_chr15 and Pf_chr19), *I. punctatus* (Ip_chr2, Ip_chr19 and Ip_chr22) and *S. meridionalis* (Sm_chr14, Sm_chr18 and Sm_chr27), and four of *P. hypophthalmus* (Ph_chr13, Ph_chr18, Ph_chr24 and Ph_chr30) (Fig. 3E). The fourth cluster was the simplest, containing two chromosomes from *P. ussuriensis* (Pu_chr4 and Pu_chr23), *I. punctatus* (Ip_chr7 and Ip_chr27), *S. meridionalis* (Sm_chr6 and Sm_chr26), and *P. hypophthalmus* (Ph_chr4 and Ph_chr25), and one from *P. fulvidraco* (Pf_chr1) (Fig. 3F). Based on these evolutionary chromosome clusters and the syntenic relationships between channel catfish and zebrafish chromosomes,^106^ the formation of chromosomes was deduced for *P. ussuriensis* (Fig. 3G).

### 3.4. Identification of the sex-determination region

Through homologous mapping of SNP-containing 2b-RAD tags from the linkage map to the assembled genome, each LG of the genetic linkage map corresponded to a chromosome of the genome assembly (Table 2). The results of QTL-mapping indicated that sex-related QTLs were all located on LG1 (chr08 of the genome assembly). A total of 546 sex-related QTLs with an LOD range of 5.24–2,247.55 (the chromosome-wide LOD threshold was 5.2) were finally identified with 539 of which being genome-wide level QTLs (the LOD threshold was 7.0). The PVE values of these QTLs varied from 15.0% to 100.0%, and PVE values of 317 QTLs were higher than 90.0%, among which 143 had PVE values of 95.0–99.9% and 62 owned a PVE value of 100.0% (Supplementary Table S6). The 62 sex-related QTLs with the PVE value of 100.0% were located on an interval of 69.93 cM (from 11.56 to 81.49 cM) on LG1 of the male genetic linkage map (Supplementary Fig. S2).

The 62 sex-related QTLs were then compared to the assembled genome, and 57 of them were mapped on the genome assembly, with 55 (96.5%) of which being mapped on a 15.76 Mb region (from 7.20 to 22.96 Mb) of chr08 (Supplementary Table S6). In one of our previous study, 327 potential male-specific tags were identified through 2b-RAD sequencing,^64^ and they were also located in the assembled genome. The results showed that only one of the 327 tags were not mapped, and 314 (96.3%) of the mapped 326 tags were all located at a 16.83 Mb region (from 6.90 to 23.73 Mb) of chr08 (Supplementary Table S7). The previously reported male-specific microsatellite *PuGT54*^21^ was located at the position of 19.48 Mb, and the male-specific sequence^19^ was located at the region of 8.36–8.37 Mb on chr08 (Supplementary Table S7). All of the above regions were then integrated and formed a final potential sex-determination region of 16.83 Mb (from 6.90 to 23.73 Mb) on chr08.

### 3.5. Screening of potential sex-determining genes

According to the results of gene prediction on chr08, a total of 554 genes were identified in the sex-determination region (Supplementary Table S8). Among the 554 predicted genes, 510 were annotated in at least one of the five public databases (Supplementary Table S8). The results of KEGG annotations showed that 353 of the 554 genes could be classified into 260 pathways (Supplementary Table S9), among which some sex-related pathways, such as TGF-beta signaling pathway, Wnt signaling pathway, estrogen signaling pathway and ovarian steroidogenesis, were identified (Supplementary Table S9). Through referring to the comparative transcriptome data between males and females,^100^ 181 of the 554 predicted genes were found as DEGs with 85 being highly expressed in males and 96 highly expressed in females (Supplementary Table S8). The 554 genes were then compared with DEGs between males and pseudofemales,^100^ and 150 DEGs were found, among which 135 coincided with DEGs between males and females and had the same expression mode (Supplementary Table S8). Therefore, the 135 DEGs, with 65 highly expressed in males and 70 highly expressed in females/pseudofemales, may contain potential sex-determining genes. Results of annotations showed that 130 of the 135 genes could be found as homologs in at least one public database (Supplementary Table S8).

Through GO analysis, 86 of the 135 DEGs were classified into 35 level2 GO terms, among which, 13 terms showed sex-specific expression trends with seven in males and six in females (Supplementary Fig. S3). The seven male-specific terms contained eight genes including Chr08.223 (nuclear receptor subfamily 4 group A member 2, *nr4a2*), Chr08.261 (insulin-like growth factor-binding protein 5, *igfbp5*), Chr08.262 (insulin-like growth factor-binding protein 2-B, *igfbp2b*), Chr08.312 (indian hedgehog B protein, *ihhb*), Chr08.326 (neuropilin-2, *nrp2*), Chr08.368 (interferon-induced helicase C domain-containing protein 1, *ifih1*), Chr08.375 (bromodomain adjacent to zinc finger domain protein 2B, *baz2b*), and Chr08.409 (homeobox protein Hox-D3, *hoxd3a*), while the six female-specific terms contained six genes, which were Chr08.269 (gap junction gamma-1 protein, *gjc1*), Chr08.320 (tubulin alpha chain, *tba*), Chr08.430 (rap guanine nucleotide exchange factor 4, *rapgef4*), Chr08.435 (histone acetyltransferase type B catalytic subunit, *hat1*), Chr08.470 (Cis-aconitate decarboxylase, *acod1*), and Chr08.475 (general transcription factor IIF subunit 2, *gtf2f2*) (Supplementary Table S10). The results of KEGG analysis showed that 88 DEGs were classified into six class 1 and 33 class 2 terms involving 126 pathways (Supplementary Fig. S4 and Table S11), and PI3K-Akt signaling pathway, aldosterone synthesis and secretion and cholesterol metabolism were the top three sex-related pathways (Supplementary Table S11). Through integrating the results of GO and KEGG enrichments, 16 candidate sex-determining genes, including ten male-highly expressed and six female-highly expressed genes, were finally identified (Table 5).

**Table 5.** Candidate sex-determining genes in the sex-determination region of *Pseudobagrus ussuriensis*

Gene	log2(m/f)	NR_Annotation	Sex-related KEGG pathway
Chr08.223	8.3753	Nuclear receptor subfamily 4 group A member 2 (*nr4a2*)	Aldosterone synthesis and secretion
Chr08.303	8.5218	Low-density lipoprotein receptor-related protein 2 (*lrp2*)	Hedgehog signaling pathway; cholesterol metabolism
Chr08.358	−2.4828	Acyl-CoA-binding protein (*acbp*)	PPAR signaling pathway
Chr08.368	4.4330	Interferon-induced helicase C domain-containing protein 1 (*ifih1*)	RIG-I-like receptor signaling pathway
Chr08.399	4.3033	Caspase-8 (*casp8*)	TNF signaling pathway
Chr08.409	10.8953	Homeobox protein Hox-D3 (*hoxd3a*)	–
Chr08.428	−7.3091	Cell division cycle-associated protein 7 (*cdca7*)	–
Chr08.520	−4.4996	Serine/threonine-protein phosphatase 2A regulatory subunit B″ subunit beta (*ppp2r3b*)	PI3K-Akt signaling pathway
Chr08.527	−4.2231	Cell division cycle protein 16 homolog (*cdc16*)	Cell cycle; oocyte meiosis; progesterone-mediated oocyte maturation
Chr08.566	3.5367	Sterol 26-hydroxylase, mitochondrial (*cyp27a1*)	PPAR signaling pathway; cholesterol metabolism
Chr08.570	3.4892	Collagen alpha-2(IV) chain (*col4a2*)	PI3K-Akt signaling pathway
Chr08.571	4.4797	Collagen alpha-2(IV) chain (*col4a2*)	PI3K-Akt signaling pathway
Chr08.572	3.5372	Collagen alpha-1(IV) chain (*col4a1*)	PI3K-Akt signaling pathway
Chr08.622	3.2926	3-Beta-hydroxysteroid dehydrogenase/Delta 5—> 4-isomerase (*hsd3b*)	Aldosterone synthesis and secretion; steroid hormone biosynthesis; ovarian steroidogenesis; cortisol synthesis and secretion
Chr08.746	−4.8826	Testis-expressed protein 30 (*tex30*)	–
Chr08.760	−4.7110	Frizzled-5 (*fzd5*)	Wnt signaling pathway

## 4. Discussion

In the present study, a chromosome-level genome of *P. ussuriensis* was successfully assembled. The genome size of *P. ussuriensis* was 741.97 Mb, containing 26 chromosomes, which was identical to the previous karyotypic results of this fish.^107^ The size of the *P. ussuriensis* genome was similar to those of reported catfish species (Table 4), and was closest to those of two Siluridae fishes: 738.90 Mb in *S. meridionalis*^35^ and 744.12 Mb in *S. asotus.*^102^ When compared with genome sizes of three reported Bagridae fishes,^33^^,^^36^^,^^38^ the genome size of *P. ussuriensis* was closest to that of *P. fulvidraco* (Table 4). The GC content in the genome of *P. ussuriensis* was similar to other catfishes (Table 4). In fact, the GC content of Siluriformes species seems very stable, with a percentage of about 39% (Table 4). Through three prediction methods, 24,075 protein-coding genes were identified in the *P. ussuriensis* genome, which was similar to those of reported Bagridae fishes and more than catfishes in families of Sisoridae, Siluridae, Clariidae, Ictaluridae, and Pangasiidae (Table 4). Therefore, this result indicated that Bagridae fishes may contain more genes than the families mentioned above. Moreover, the two Bagridae fishes *P. ussuriensis* and *P. fulvidraco* also had more unique genes and gene families than *S. meridionalis* (Siluridae), *I. punctatus* (Ictaluridae), and *G. maculatum* (Sisoridae) (Supplementary Table S5). The more frequent expansions and contractions of gene families may be the key reasons for the larger number of genes and more unique genes and gene families in Bagridae fishes than catfishes of other families (Fig. 2C).

Four different genera of Bagridae, including *Pseudobagrus*, *Pelteobagrus*, *Leiocassis*, and *Hemibagrus*, have been reported to occur in East Asia.^108^ The results of phylogenetic analyses showed that *P. ussuriensis* had the closest relationship to *P. fulvidraco*, and these two species separated in ∼11.04 Mya (Fig. 2B). According to previously reported studies, separation times were 41.73 Mya between *H. wyckioides* and *P. fulvidraco*,^38^ and 26.2 Mya between *L. longirostris* and *P. fulvidraco*.^36^ Therefore, among the four genera of the Bagridae family, *Pseudobagrus* and *Pelteobagrus* had the closet relationship. The phylogenetic relationships within the four genera of Bagridae are still equivocal and controversial. Based on skeleton characters, Dai *et al*. pointed out that *Leiocassis* was the most original genus, followed by *Pseudobagrus* and *Pelteobagrus*, and *Hemibagrus* was the most developed genus.^109^ While *Hemibagrus* was believed to be at a basal position based on mitochondrial DNA sequences, and *Pseudobagrus*, *Pelteobagrus*, and *Leiocassis* were found to be polyphyly in many studies.^108^^,^^110^^,^^111^ Moreover, Ku *et al.* proposed that *Hemibagrus* and *Pseudobagrus* were the only two valid genera of East Asian bagrids, and the Chinese species assigned to *Pelteobagrus* and *Leiocassis* should be considered members of the genus *Pseudobagrus*.^108^ The assembled genome of *P. ussuriensis* obtained in this study will be helpful for further investigations on classifications of Bagridae species at the whole genome level.

Previous studies have found that majority fishes from families of Bagridae, Sisoridae, Horabagridae, and Amblycipitidae were involved in a predominant Asian group, which were called the ‘Big Asia’.^111^^,^^112^ The phylogenetic tree obtained in this study also accorded with this opinion, as Bagridae and Sisoridae formed a clade with strong supports (Fig. 2A). The estimated divergent times of Bagridae and Sisoridae in this study (Fig. 2B) also conformed to the viewpoint that these two major families within the ‘Big Asia’ clade occurred just after the K-Pg boundary (<66 Mya).^111^ The ‘Big Asia’ clade in this study was closer to the family Ictaluridae than Siluridae, and the similar phenomenon has also been reported in previous studies.^38^^,^^111^ It has been reported that, although Ictaluridae is endemic in North American, it can be connected with Asian catfishes through its close relative in Asia, the Cranoglanididae family.^111–113^ Hardman deduced that the ancestor of Ictaluridae speciated from the ancestor of Cranoglanididae, and have invaded freshwaters of North America at the close of the Cretaceous through northeastern.^113^ While Siluridae has been reported to be a monophyletic family, and its connections to other catfish families were still unresolved.^111^^,^^113^ Additionally, *P. ussuriensis* and other catfishes formed the Siluriformes clade, and showed a closer relationship with Characiformes than Cypriniformes in the Ostariophysi, which coincided with results of previous studies.^34^^,^^38^^,^^114^

It has been reported that the ancient chromosome number of bony fish was 12 pairs, and formations of the present various chromosome numbers were the results of whole genome duplication, chromosomal fission/fusion, and intra/inter-chromosomal rearrangements.^115–118^ In Siluriformes species, chromosome numbers varied from 2n = 24 to 2n = 100,2 and 2n = 58 was deduced to be the ancestral number.^5^ Our results also support this deduction, as the two species *S. meridionalis* and *I. punctatus*, both of which were at earlier divergent statuses of the phylogenetic tree (Fig. 2A), had a haploid chromosome number of 29. As both of the two close-related fishes (in orders of Characiformes and Cypriniformes) of Siluriformes had 25 chromosome pairs, the ancient 29 chromosome pairs of Siluriformes may evolved from 25 chromosome pairs. The chromosomal syntenic relationships between *I. punctatus* and zebrafish showed that each of Ip_chr2 and Ip_chr12 corresponded to a sole zebrafish chromosome instead of two,^106^ therefore, *I. punctatus* may preserve more ancestral chromosome structures than *S. meridionalis* and *P. hypophthalmus* (Fig. 3D and E). Using chromosomes of *I. punctatus* as the ancestral type, chromosome number changes among five catfish species were analyzed, which mainly involved four chromosome clusters (Fig. 3C−F). In the first cluster, *S. meridionalis* and *P. hypophthalmus* preserved the original chromosomes, Pu_chr1 fused Ip_chr10 and Ip_chr20, while Pf_chr2 fused Ip_chr10 and Ip_chr21 (Fig. 3C). In the second cluster, Ip_chr12 was broken into two segments, one of which solely formed a new chromosome, and the other one fused with Ip_chr11 in *S. meridionalis* and *P. hypophthalmus*; in *P. ussuriensis*, Pu_chr3 fused Ip_chr25 and one segment of Ip_chr12, Pu_chr5 fused Ip_chr11 and the other segment of Ip_chr12, Pu_chr2 fused Ip_chr13 and Ip_chr29; in *P. fulvidraco*, Pf_chr5 fused Ip_chr11 and an Ip_chr12 segment, Pf_chr7 fused Ip_chr29 and the other Ip_chr12 segment (Fig. 3D). In the third cluster, chromosomal fission was occurred on Ip_chr2, and formed two new chromosomes in *P. hypophthalmus*, which was also the direct causation of 30 chromosomes in *P. hypophthalmus*; in *S. meridionalis*, one segment of Ip_chr2 solely formed a new chromosome and the other segment fused with Ip_chr19; while in *P. ussuriensis* and *P. fulvidraco*, an Ip_chr2 segment formed a new chromosome solely and the other segment fused with Ip_chr22 (Fig. 3E). In the fourth cluster, Ip_chr7 and Ip_chr27 fused in *P. fulvidraco* but not in the other four species (Fig. 3F). Based on these chromosome clusters and chromosomal syntenic relationships between *I. punctatus* and zebrafish,^106^ the formation of the haploid 26 chromosome number in *P. ussuriensis* was inferred, which experienced a process of 25–29–26, and chromosomal fissions and fusions were dominant events for chromosome number changes as reported in other studies.^118–120^

Sex chromosomes are the most concerned chromosomes in teleosts, and origins and evolutions of them are considered as central topics in evolutionary genetics.^14^ However, as sex chromosomes are morphologically undifferentiated and homomorphic in most teleosts, identifications of sex chromosomes should rely on sex-determining genes or sex-specific molecular markers. QTL-mapping is a useful method to find sex-related loci and identify potential sex chromosomes, which has been successfully used in many fishes.^25^^,^^26^^,^^121^ In the present study, 546 sex-related QTLs including 62 QTLs with a PVE value of 100.0% were detected on LG1 of the *P. ussuriensis* genetic linkage map, which corresponded to chr08 of the assembled genome. The number of identified sex-related QTLs was much more than that of many fishes.^26^^,^^121^^,^^122^ In many previously reported fishes, sex-related QTLs were located at short regions of LGs, such as 5.70 cM in *Pundamilia pundamilia*,^121^ 3.86 cM in *Hypophthalmichthys nobilis*,^123^ and 0.40 cM in *Channa argus*,^122^ however, the sex-related QTL region in this study was quite large, which spanned 69.93 cM of LG1. Although large sex-related QTL regions have also been identified in other fishes, including 66.03 cM in channel catfish,^106^ 70.88 cM in *Scophthalmus maximus*^124^ and 124.72 cM in *Nibea albiflora*,^26^ the values of LOD and PVE of QTLs in *P. ussuriensis* were much higher. Given the larger number, higher LOD and PVE values and wider spanning regions of sex-related QTLs in *P. ussuriensis*, the differentiation level of sex chromosomes may be higher than fishes mentioned above.

These QTLs were mapped to the assembled chromosome-level genome of *P. ussuriensis*, and located at a 15.76-Mb region on chr08. Furthermore, previously identified male-specific 2b-RAD tags,^64^ the male-specific microsatellite *PuGT54*21 and a male-specific sequence^19^ were also located on chr08, forming a combined potential sex-determination region of 16.83 Mb (from 6.90 to 23.73 Mb). It has been reported that to increase the association of sexually antagonistic alleles with the sex-determining locus, recombination on sex chromosomes was usually reduced,^125^ therefore, sex-biased genetic polymorphisms can be found on sex chromosomes.^27^ The results in the present study also support this viewpoint, as hundreds of male-specific loci concentrated in the potential sex-determination region. As chr08 was the only chromosome that contained the potential sex-determination region, it was deduced to be the sex chromosome, to be precise, the X chromosome, of *P. ussuriensis*. Sex chromosomes were believed to be the most difficult part of the genomes to study, thus most fish genome projects have purposely avoided the heterogametic sex, even if new sequencing technologies have been used.^14^ In the present study, although the homogametic female was used to obtain the genome assembly of *P. ussuriensis*, the sex chromosome and potential sex-determination region were still identified with the help of sex-specific QTLs, tags, markers and sequences. In our future studies, the YY super-male individuals will be bred to obtain the Y chromosome sequence, which will be compared with the sequence of X chromosome obtained in this study, and the sex-determination region will be located on a more accurate segment of the Y chromosome. By then, answers of many questions, including the evolution process of sex chromosomes, whether there were male-specific sex-determining genes on the Y chromosome, whether some male-specific sites/genes on chr08 were possible fast-evolving sites/genes, will be revealed.

Sex-determining genes are greatly diverse in fish, and genes from classical transcription factors such as *dmy*^126^ and *sox3*,127 to genes related to TGF-β such as *gsdf*^128^ and *amh*^129^^,^^130^ and its receptor *amhr2*,131 or steroidogenic pathway-related genes such as *hsd17b1*132 have been identified as sex-determining genes. Among these genes, *dmrt1*, *amh*, *sox3*, and *gsdf* have conserved roles in the vertebrate sex-determination pathway, and are called ‘usual suspects’.^133^ However, in recent years, more ‘newcomers’ of sex-determining genes, which had no previously known sex determination functions, have been identified, such as *Paics* in tilapia,^10^*bcar1* in channel catfish,^134^ and *sdY* in salmonids.^135^ In the present study, 554 genes were identified in the potential sex-determination region of *P. ussuriensis*. However, none of the sex-determining genes that previously reported in other fishes were detected in this region. In fact, homologs of these genes were not even located on the deduced sex chromosome chr08, which indicated that the sex-determination mechanism of *P. ussuriensis* may be complex. Although comparisons between transcriptomes of female and male gonads indicated that *dmrt1* and *gdf9* may play essential roles in sex determination of *P. ussuriensis*,^100^ the sex-determining genes should be reconsidered, since the two genes were, respectively, mapped on chr10 and chr15, instead of on the sex-determination region of chr08. Expression data of the 554 genes from the sex-determination region were extracted from a transcriptomic study,^100^ in which transcriptomes between males and females, and between males and pseudofemales were compared. Finally, 135 of the 554 genes were identified as DEGs. Sixteen potential sex-determining genes were screened out, but their roles in sex determination of *P. ussuriensis* were still unknown. Studies on expression patterns of these candidate genes at different developmental stages of the gonads will be performed in our following work, which may provide supportive evidences to determine truly potential candidate sex-determining genes. Moreover, further functional analyses will be performed for these candidate sex-determining genes, moreover, other ‘newcomers’ will also be considered in our future studies.

In conclusion, a chromosome-level genome was assembled and annotated for female *P. ussuriensis*. Chromosomal evolutions between *P. ussuriensis* and other catfishes were analyzed with four evolutionary chromosome clusters being identified, and the formation of the *P. ussuriensis* chromosome number was deduced. A sex-determination region was focused on chr08, which was predicted as the X chromosome, and sixteen genes in this region were considered as candidate sex-determination genes. The chromosome-level genome obtained herein will provide basic data for genetic and genomic studies of *P. ussuriensis*, moreover, results of chromosomal evolution and sex-determination analyses will be helpful to analyze genomic evolution of Siluriformes species and clarify the sex-determination mechanism of *P. ussuriensis*.

## Supplementary data

Supplementary data are available at DNARES online.

## Supplementary Material

dsac028_Supplementary_Data

## Data Availability

The raw data of genome and RNA sequencing have been deposited in the Sequence Read Archive (SRA) database under the BioProject PRJNA813030, and the genome assembly is available at GenBank under the BioProject PRJNA810258.
