# Supplementary figures and images for: Insights into chromosomal evolution and sex determination of Pseudobagrus ussuriensis (Bagridae, Siluriformes) based on a chromosome-level genome
Source: DNA Res. 2022 Jul 21;29(4):dsac028. doi: 10.1093/dnares/dsac028 (PMC9358014; doi:10.1093/dnares/dsac028)

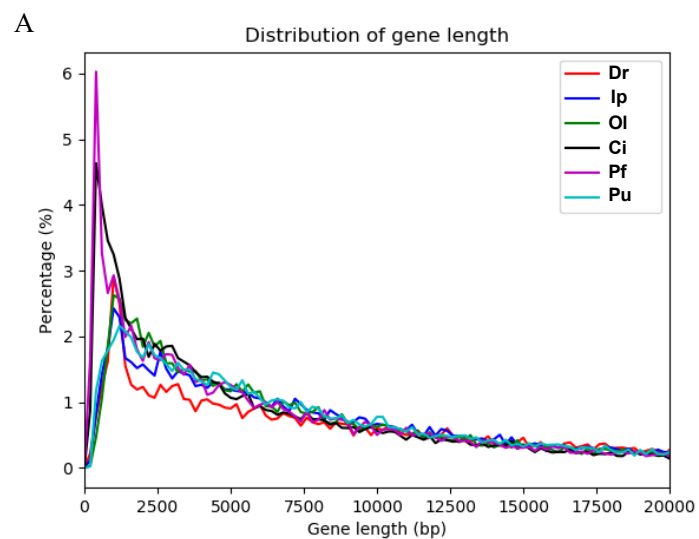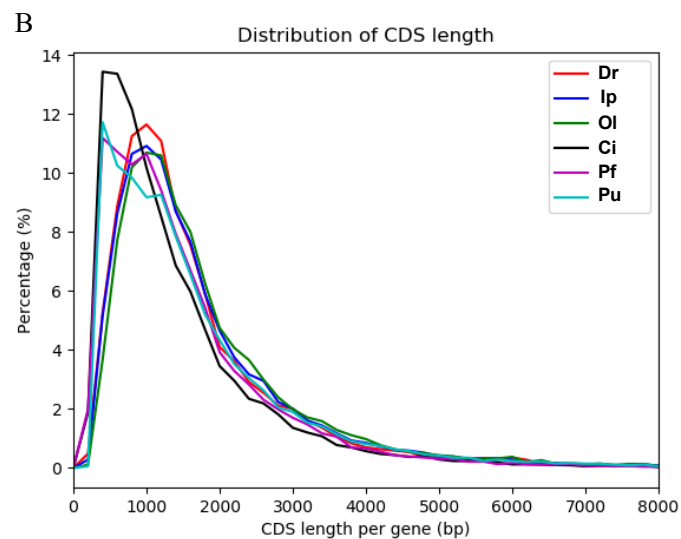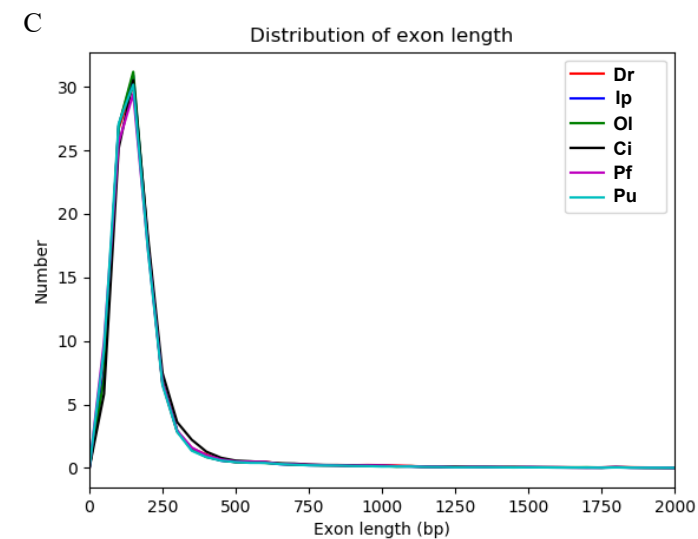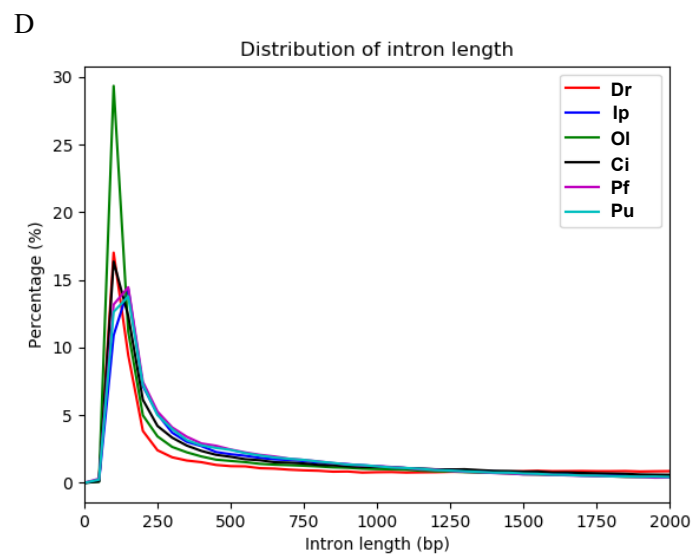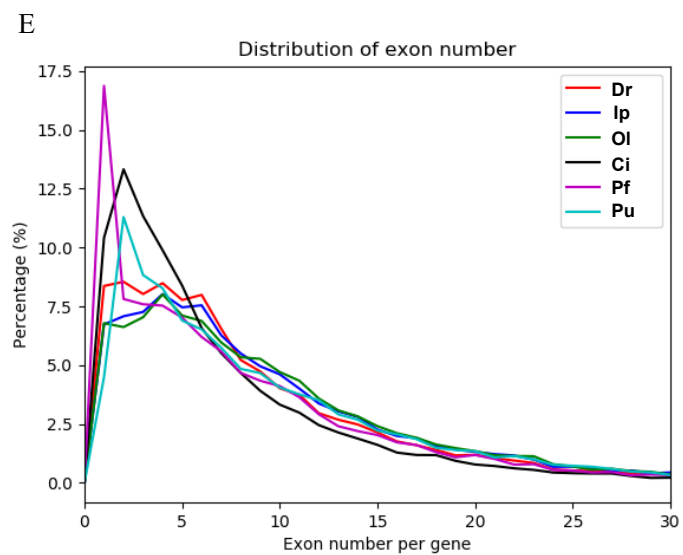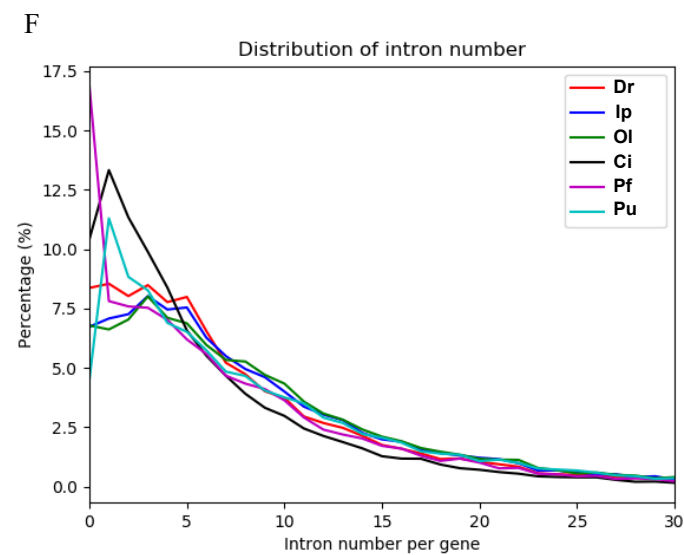

Supplement: dsac028_Supplementary_Data [file dsac028_supplementary_data.zip › dsac028_Supplementary_Data/Fig.S1.pdf]

1

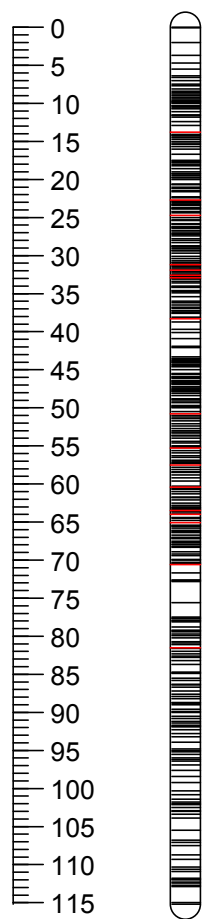

Supplement: dsac028_Supplementary_Data [file dsac028_supplementary_data.zip › dsac028_Supplementary_Data/Fig.S2.pdf]

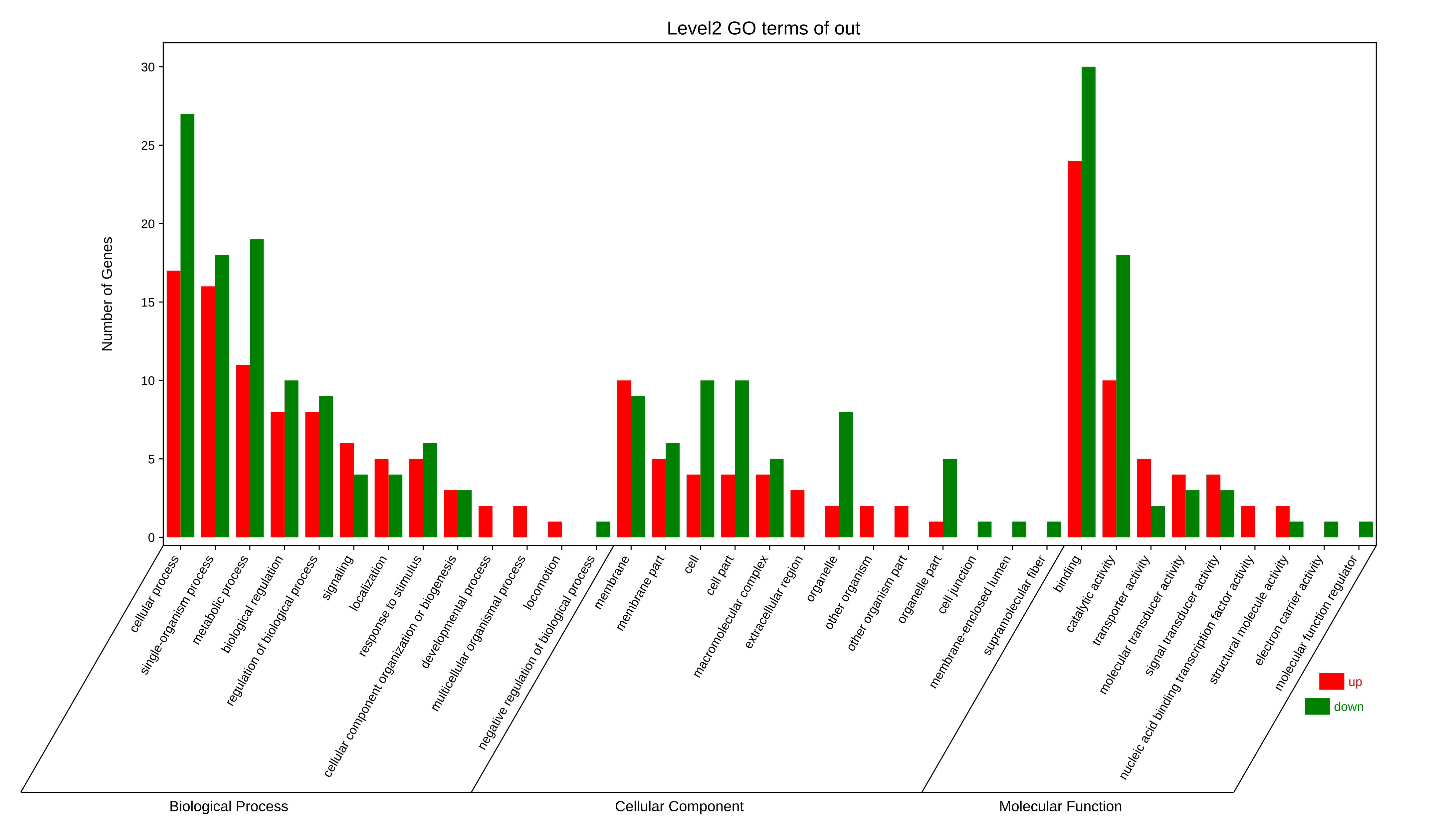

Supplement: dsac028_Supplementary_Data [file dsac028_supplementary_data.zip › dsac028_Supplementary_Data/Fig.S3.png]

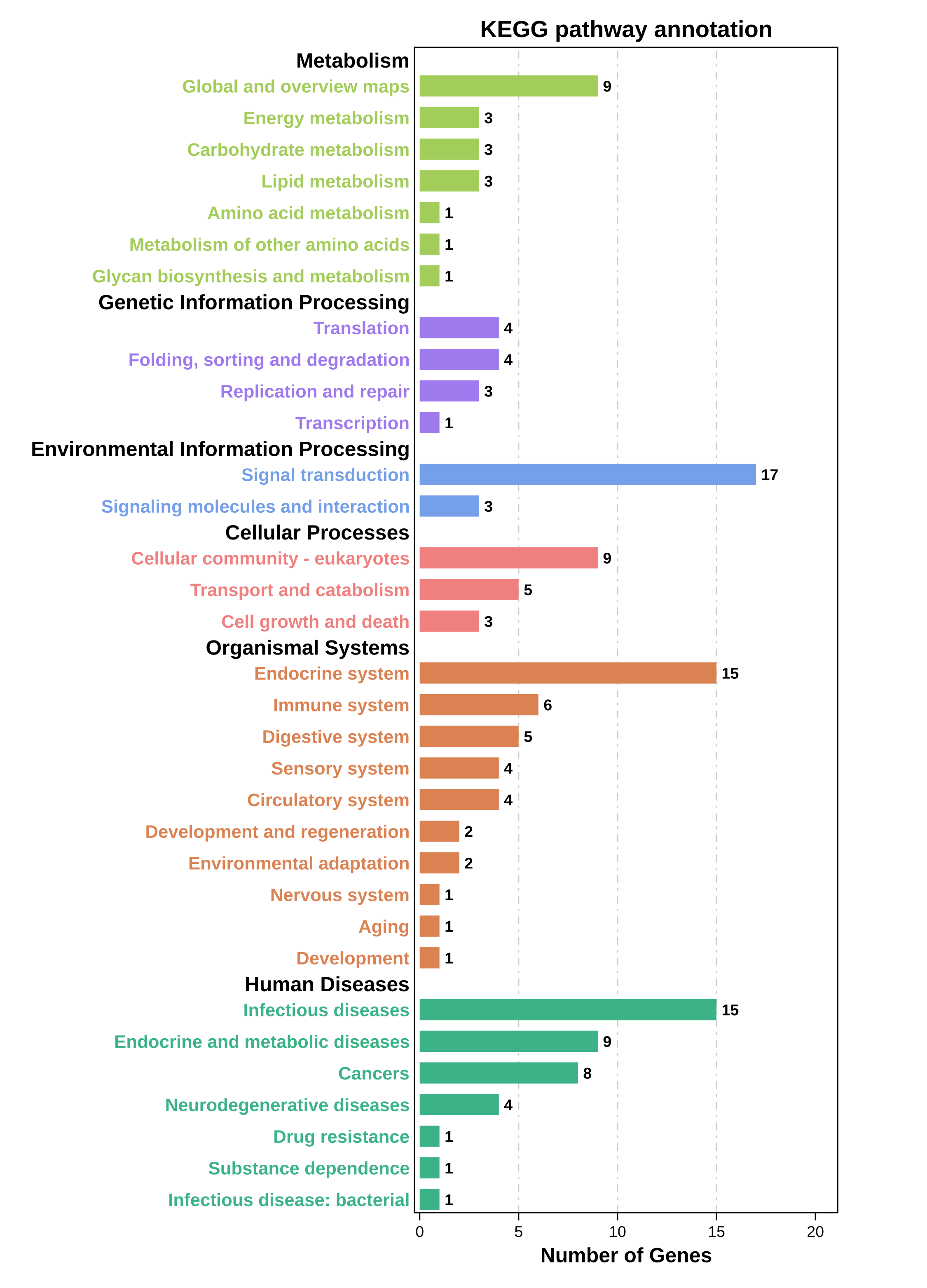

Supplement: dsac028_Supplementary_Data [file dsac028_supplementary_data.zip › dsac028_Supplementary_Data/Fig.S4.png]
